# BEOL‐Compatible 4F^2^ Oscillator Using Vertical InGaAs Biristor for Highly Scalable Monolithic 3D Ising Solver

**DOI:** 10.1002/smll.202406822

**Published:** 2024-10-21

**Authors:** Joon Pyo Kim, Hyun Wook Kim, Jaeyong Jeong, Juhyuk Park, Song‐Hyeon Kuk, Jongmin Kim, Jiyong Woo, Sanghyeon Kim

**Affiliations:** ^1^ School of Electrical Engineering Korea Advanced Institute of Science and Technology (KAIST) 291 Daehak‐ro, Yuseong‐gu Daejeon 34141 Republic of Korea; ^2^ School of Electronic and Electrical Engineering Kyungpook National University (KNU) 80 Daehak‐ro, Buk‐gu Daegu 41566 Republic of Korea; ^3^ Device Technology Division Korea Advanced Nano Fab Center (KANC) 109 Gwanggyo‐ro, Yeongtong‐gu Suwon 16229 Republic of Korea

**Keywords:** biristor, combinatorial optimization, Ising solver, MaxCUT, oscillator

## Abstract

Ising solvers are important for efficiently addressing non‐deterministic polynomial‐time (NP)‐hard combinatorial optimization problems (COPs), where scalability and compactness are crucial for practical applications. In this study, an experimental demonstration of an oscillator‐based Ising solver employing a highly scalable 4F^2^ InGaAs bi‐stable resistor (biristor) is presented. It is first explored the oscillation behavior of the InGaAs biristor, establishing that classical Ising spins can be emulated using the sub‐harmonic injection locking (SHIL) technique. Furthermore, capacitive and resistive coupling between two coupled InGaAs biristors is demonstrated, leading to out‐of‐phase and in‐phase coupling, respectively. Employing this foundational technology, it is experimentally achieved a solution to the MaxCUT problem with the InGaAs biristor‐based Ising solver, supplemented by simulation‐based behavior evaluations. This emerging device architecture offers a viable pathway to surmount the scaling limitations faced by present hardware‐based Ising solvers, representing a significant step forward in the development of efficient, scalable solutions for complex optimization challenges.

## Graphical abstract

Ising solvers are important for efficiently addressing NP‐hard combinatorial optimization problems. In this study, an experimental demonstration of an oscillator‐based Ising solver employing a highly scalable 4F^2^ InGaAs bi‐stable resistor (biristor) is presented. This emerging device architecture offers a viable pathway to surmount the scaling limitations faced by present hardware‐based Ising solvers.

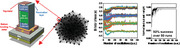

## 1 Introduction

Efficiently solving combinatorial optimization problems (COPs) is crucial, given their extensive applications in critical domains such as artificial intelligence, finance, drug discovery, cryptography, telecommunications, and more.^[^ ^1^ ^]^ These problems, classified as nondeterministic polynomial‐time hard (NP‐hard), present significant computational challenges, with the difficulty of finding optimal solutions increasing exponentially with problem size.^[^ ^2^ ^]^ The Ising model, originating from statistical mechanics, excels at leveraging the inherent structure of complex problems to converge toward states that minimize the objective function efficiently. This capability enables it to find near‐optimal solutions effectively. The Ising model introduces a global energy function, the Ising Hamiltonian *H*(σ), which is a network of *n* binary spins *σ* ∈(+ 1, − 1) to represent the spin up or down, connected by *J*_ij_ to represent the interaction coefficient between spins *σ*_i_ and *σ*_j_. The quantity of spins and the graph topologies of the spin network directly influence the scale and complexity of COPs solvable by the Ising model. The model seeks the spin configuration that minimizes the Ising Hamiltonian energy function, represented as follows^[^ ^3^ ^]^:

(1) $$H\;\left(\sigma \right)=-\;\sum_{1\leq i\leq j\leq n}{J_{i}}_{j}\sigma_{i}\sigma_{j}\;-\;\sum_{i=1}^{n}h_{i}\sigma_{i}$$

where *h*_i_ denotes the interaction of the external magnetic field on each spin. While heuristic algorithms offer a means to navigate the state space toward nearly optimal solutions, they rely on traditional von Neumann architecture, leading to exponential increases in computing time and power consumption as problem complexity grows.^[^ ^4^ ^]^ This highlights the need for innovative computational strategies beyond the capabilities of conventional processor architectures.

Ising machines, specialized hardware designed to tackle COPs by mapping them onto the Ising model, represent a promising solution.^[^ ^5^ ^]^ Various technologies, including superconducting qubits,^[^ ^6^ ^]^ coherent lights,^[^ ^7^, ^8^ ^]^ and field‐programmable gate arrays (FPGAs),^[^ ^9^ ^]^ have been explored. However, quantum computers require ultra‐low temperature operation, making them cost‐prohibitive. Optical coherent Ising machines, despite their performance, necessitate lengthy fiber ring cavities for temporal multiplexing, adding complexity and scalability challenges. FPGAs, though practical for computational tasks, face scalability issues that limit their application in solving large‐scale COPs crucial for real‐world scenarios.

In this context, the oscillator‐based Ising machine (OIM), introduced by Wang et al.,^[^ ^10^ ^]^ utilizes the dynamics of coupled oscillators, with each acting as a spin node represented by the oscillator's phase. If a continuous dynamical system is created with the network of coupled oscillators under sub‐harmonic injection locking (SHIL), the system can naturally find the ground state over time. The intrinsic ability of these oscillators to synchronize and evolve toward minimal energy states embodies the solution‐seeking process of the Ising problem. From this concept of the Ising solver, OIMs using LC oscillators^[^ ^11^ ^]^ or complementary metal‐oxide‐semiconductor (CMOS) ring oscillators^[^ ^12^ ^]^ were reported; however, these approaches face challenges in terms of scalability and large footprint area. Furthermore, compact devices such as insulator‐to‐metal transition (IMT) material‐based oscillators were reported.^[^ ^13^ ^]^ However, as the device scales to the nanoscale, the instability of the IMT leads to device variability, which limits its use as a large‐scale Ising solver as the frequency dispersion may significantly impact the system performance.^[^ ^14^ ^]^

**Figure 1:**
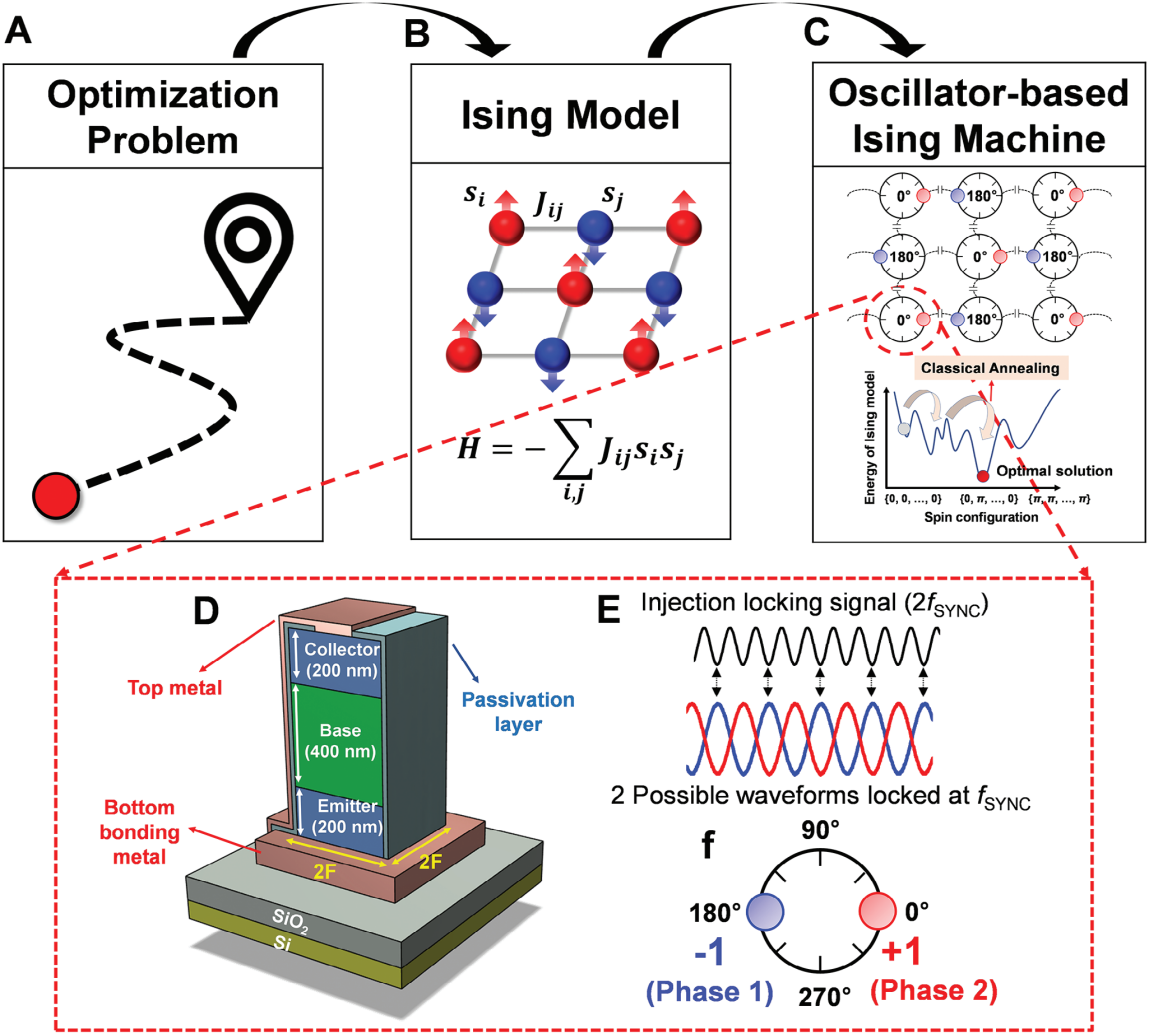
a) Illustration of a generic optimization problem. b) Mapping the optimization problem to the Ising model, which introduces the Ising Hamiltonian *H*. c) Mapping the Ising model to the coupled oscillator‐based hardware. The coupled oscillator network finds the global minimum of the Hamiltonian solution space through classical annealing. d) The 3D schematic structure of the oscillator in the Ising solver, which is made up of a vertical single crystalline npn biristor. The collector, base, and emitter of the biristor are each 200, 400, and 200 nm, respectively, and are stacked on a SiO_2_/Si substrate. Notably, the cell size is 4F^2^. e) When the frequency of the injection locking is at 2*f*_SYNC_, the oscillator locks to the frequency of *f*_SYNC_. f) Bi‐phase locking is shown with the sub‐harmonic injection locking.

In this paper, we first demonstrated a highly scalable Ising solver consisting of a back end‐of‐line (BEOL) compatible, compact vertical bi‐stable resistor (biristor) consisting of a single‐crystalline semiconductor, leading to significant improvements in scalability and power efficiency with a minimal device variability. The single‐crystalline structure of the biristor introduces a single‐transistor latch mechanism for achieving bi‐stabilty, leading to unique electrical characteristics in a simple vertical structure. This simplified design offers advantages over oscillators based on complex CMOS circuits, particularly in terms of reduced footprint area. Moreover, by utilizing a BEOL‐compatible oscillator, it is possible to stack oscillators in a monolithic 3D configuration, which not only enables structural scaling of the Ising machine but also reduces the parasitic effects on the oscillators caused by coupling elements and routing circuits. Additionally, due to the use of single crystalline semiconductor junctions, the biristor‐based oscillator is expected to be more reliable than other exotic material‐based devices, maintaining stable operation even at the nanoscale. Schematics in **Figure** **1**a‐e illustrate the concept of this work. Various real‐world COPs can be converted to the Ising model, which features coupled spin networks of a graph, and the cost function of this model is represented by the Ising Hamiltonian *H*. Such an Ising model can be directly mapped to the OIM with a network of coupled biristors. This configured Ising dynamical hardware solver evolves over time in a temperature‐dependent manner, moving toward an energy minimization state through an additional annealing process. Each spin of the OIM is characterized by a phase of a vertical biristor‐based oscillator composed of a simple single‐crystalline InGaAs semiconductor npn junction. Because the biristor exhibits a bi‐stable resistance region between latch‐up and latch‐down voltage, voltage oscillation is observed with an appropriate injected source current.^[^ ^15^ ^]^ Since this device relies on single‐crystalline semiconductor junctions, it offers consistent and stable operation, a crucial aspect for the reliability of the Ising machines. Additionally, we applied the injection locking techniques to represent the spin state with the biristor, which needs to binarize the phase of the oscillators.^[^ ^16^ ^]^ When a suitable synchronization signal (SYNC) with a frequency similar to the natural frequency of the biristor‐based oscillator (*f*_SYNC_) is applied to the biristor, injection locking occurs. When this *f*_SYNC_ is increased by a factor of *S*, the oscillator locks to one of *S* possible phases that are 360/*S*° apart. In this work, each oscillator has two states, “spin‐up” and “spin‐down”, with *S* = 2 being used. To couple each oscillator, capacitive coupling was utilized. It was observed that the constructed Ising machine prototype effectively solves simple MaxCUT problems through parallel updates. Additionally, we expanded our Ising machine through phase macro modeling and confirmed, using a proper additional annealing scheme through injection locking, that the proposed Ising solver can solve larger MaxCUT problems more efficiently and with greater scalability compared to others.

## 2 Results and Discussion

### 2.1 InGaAs Biristor‐Based Oscillator

**Figure 2:**
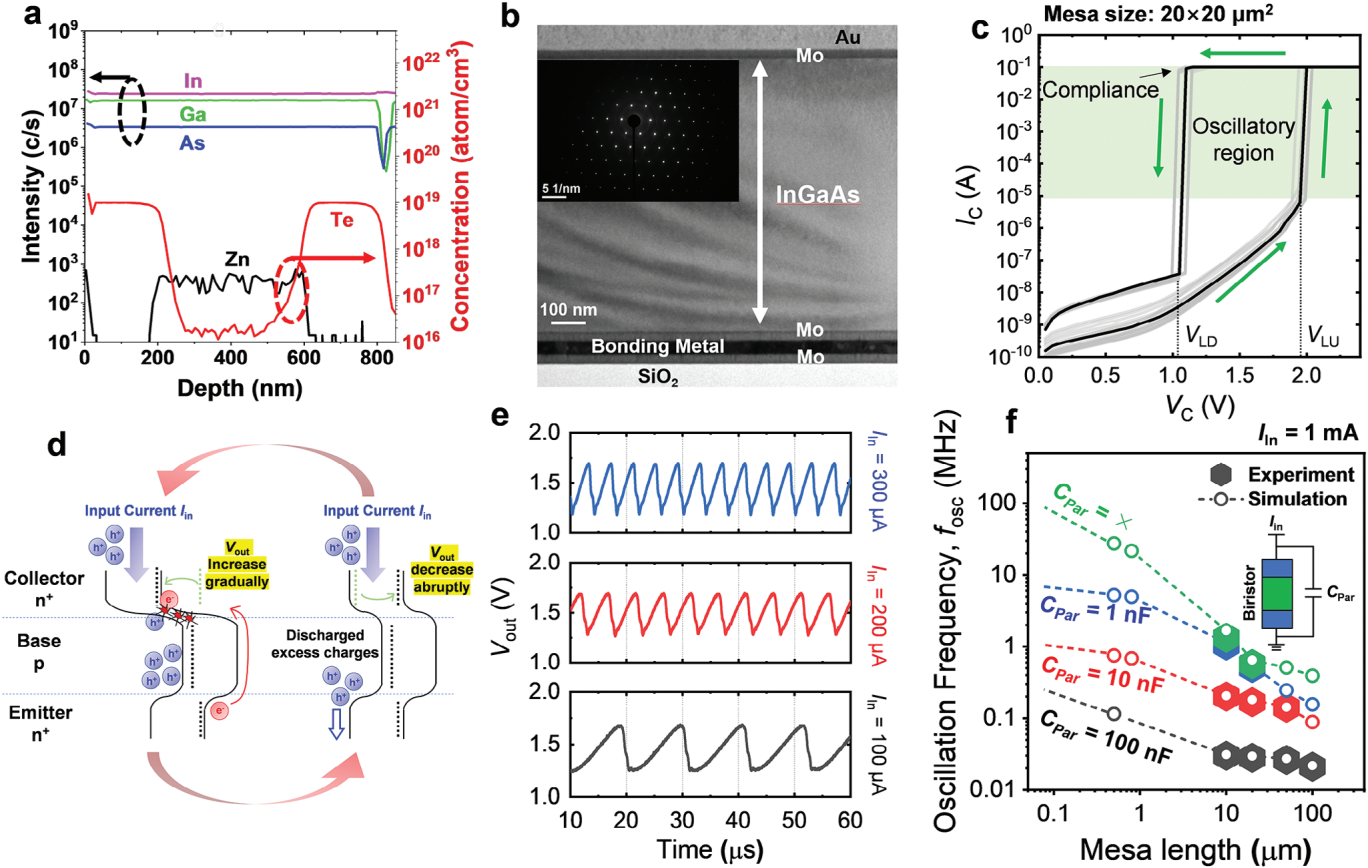
a) Secondary ion mass spectrometry (SIMS) profile of the InGaAs layer along a vertical direction. The abrupt junction of Te doping was shown between collector, emitter, and base. b) Cross‐sectional transmission electron microscopy (TEM) image of the fabricated vertical InGaAs biristor. The inset shows an electron diffraction image of the InGaAs layer, showing the high crystal quality of the InGaAs layer. c) Hysteric DC *I*_c_–*V*_c_ characteristics of the fabricated vertical InGaAs biristors with mesa size of 20 × 20 µm^2^. d) Energy band diagram of the vertical InGaAs biristor to illustrate the principle of the voltage oscillation during the applied input current. e) Output voltage oscillation waveforms according to the different input currents. As the magnitude of the *I*_in_ increases, it can be observed that the oscillation frequency also increases. f) Experimental and simulation results of the frequency of the InGaAs biristor according to the mesa size and parasitic capacitor (*C*_Par_) during the input current of 1 mA.

Since the steep junction highly impacts the latch characteristics in the biristor, we epitaxially grew n^+^pn^+^ InGaAs with a dopant of Te.^[^ ^17^ ^]^ The floating base, which is a p‐type InGaAs layer, has a thickness of 400 nm. It is evident from the secondary ion mass spectroscopy (SIMS) in **Figure** **2**a that the emitter and collector, which are highly Te doped (≈1 × 10^19^ cm^−3^) with each of 200 nm thickness, form well‐defined abrupt junctions with the base. For the layer transfer, we introduced etch‐stop layers between the n^+^pn^+^ junction of InGaAs and InP substrates. To show the BEOL compatibility, we fabricated InGaAs biristor on SiO_2_/Si substrate using a wafer‐bonding technique. The detailed fabrication process is shown in Figure S2 (Supporting Information). Notably, the fabrication process of the InGaAs biristor occurs at temperatures below 100 °C, demonstrating excellent compatibility with BEOL processes. The cross‐sectional transmission electron microscopy (TEM) image of the fabricated bonding InGaAs biristor is shown in Figure 2b, indicating the high crystal quality of the InGaAs layer and its bonding behaviors. Figure 2c shows the measured hysteric characteristic of collector current (*I*_C_) versus collector voltage (*V*_C_) of fabricated InGaAs biristors. The current is latched up due to the impact ionization at the abrupt junction between the base and collector region and latched down when the voltage reaches back.^[^ ^18^ ^]^ The difference in latch‐up voltage (*V*_LU_) and latch‐down voltage (*V*_LD_) was ≈1.0 V, showing reliable uniformity which is important for the stable Ising solver array. Notably, the bi‐stable resistance region corresponds to a current‐forbidden region, when the input current (*I*_in_) is applied to this unstable region, the biristor shows a voltage oscillation.^[^ ^15^ ^]^ Figure 2d shows the voltage oscillation mechanism of the InGaAs biristor in terms of the band structure. When the *I*_in_ is applied to the collector of the InGaAs biristor, electrons in the emitter are injected into the base causing the impact ionization. The holes generated by the former impact ionization accumulate in the floating base and lower the barrier between the emitter and base, causing the generation of more electron‐hole pairs. The output voltage (*V*_Out_) measured in the collector increases until the *V*_Out_ exceeds the *V*_LU_ and the integrated charges flow through the base, causing the *V*_Out_ to return to *V*_LD_. This voltage oscillation is repeated as positive feedback during the current injection. Figure 2e shows the clear self‐voltage oscillation in the single vertical InGaAs biristor with various input currents. If the input current is lower than what is required for the oscillatory region, all injected charges will be recombined, causing the output voltage to become stuck at 0 V. Additionally, as the input current increases, the charging and discharging processes will be accelerated, increasing the frequency of the InGaAs biristor (*f*_osc_) as shown in Figure S3 (Supporting Information). This tendency can be represented by the equation,^[^ ^19^ ^]^

(2) $$f_{\mathrm{osc}}=\frac{I_{\mathrm{in}}}{C_{\mathrm{par}}\left(V_{\mathrm{LU}}-V_{\mathrm{LD}}\right)}\;$$

where *C*_par_ denotes the parasitic capacitance. To observe the impact of the *C*_par_ and mesa size, we measured the oscillation frequency of the biristor across various *C*_par_ and mesa sizes. As indicated by the equation, a decrease in *C*_par_ led to an increase in *f*_osc_. However, there was a limit to the frequency increase even in the absence of external *C*_par_, which is attributed to the parasitic capacitance (e.g., wiring, cable, etc.) arising from the measurement setup, estimated to be ≈700 pF as shown in Figure S4 (Supporting Information). This value has also been utilized in subsequent circuit simulation parameters. Additionally, it was observed that as the mesa size decreases, *f*_osc_ increases as shown in Figure 2f. This indicates that a reduction in mesa size leads to an increase in the impact ionization rate, suggesting that scaling down the mesa size in the future could significantly enhance the frequency of the vertical InGaAs biristor‐based oscillator. This is one of the very important advantages of InGaAs biristor, providing fast problem‐solving capability. Notably, since InGaAs has a relatively narrower bandgap, the voltage spike for InGaAs occurs at less than 2 V range, significantly lower than ≈5 V seen with the similar devices based on Si, making it far more power‐efficient in terms of energy per oscillation when used as a large‐scale Ising solver.^[^ ^20^ ^]^

### 2.2 InGaAs‐Biristor Based Ising Solver

**Figure 3:**
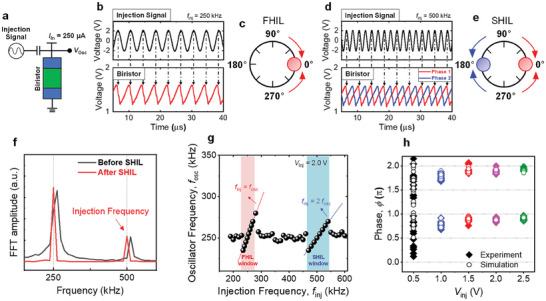
a) The measurement schematic of the injection locking in the InGaAs biristor‐based oscillator. b) Output waveforms of the injection signal and InGaAs biristor when the injection signal is tuned to the first‐harmonic injection locking (FHIL). c) The phase diagram of the InGaAs biristor during FHIL. d) Output waveforms of the injection signal and InGaAs biristor when the injection signal is tuned to the sub‐harmonic injection locking (SHIL). Notably, red and blue waveforms occur with the same probability. e) The phase diagram of the InGaAs biristor during SHIL. f) Fast Fourier Transform (FFT) plots of the InGaAs biristor before and after SHIL. g) Oscillation frequency of the InGaAs biristor (*f*_osc_) versus frequency of injection locking signal (*f*_inj_) with injection voltage (*V*_inj_) = 2.0 V. The red dashed line shows when *f*_inj_ = *f*_osc_ and the blue dashed line shows when *f*_inj_ = 2 *f*_osc_. h) Measurement and simulated results of the phases of the InGaAs biristor according to the various *V*_inj_.

To construct an OIM, the oscillators must be coupled and form a network to naturally minimize the Ising Hamiltonian. However, as the scale increases, the phases of the oscillators tend to settle discretely, making it difficult to reach lower energy states of the Ising Hamiltonian. Therefore, by introducing SHIL, the phases of the oscillators can be forced into two distinct states, altering the dynamics of the coupled oscillators and allowing the system to achieve lower energy states of the Ising Hamiltonian more effectively. When an oscillator is perturbed by an external oscillator with a similar frequency, it locks to that frequency. If the oscillator is locked by an external signal whose frequency is twice of the oscillator frequency, it settles into one of the two steady states. This phenomenon can be explained through a Generalized‐Adler equation that describes a non‐linear oscillator network perturbed by the external injection signal (see Supplementary Note 1, Supporting Information).^[^ ^21^ ^]^ The schematic for the measurement of injection locking in the InGaAs biristor‐based oscillator is presented in **Figure** **3**a. We adjusted the input current to 250 µA to attain a *f*_osc_ of ≈250 kHz. By utilizing a function generator, we introduced a sinusoidal wave with an injection capacitance (*C*_inj_) of 10 pF, ensuring that *f*_osc_ closely aligns with the injection frequency (*f*_inj_), corresponding to the phenomenon of first harmonic injection locking (FHIL). As shown in Figure 3b, by using the injection signal's peak at phase = 0° as a reference point, it is observable that the biristor's frequency locks to the injection signal near the 0° (Figure 3c). Moreover, setting 2*f*_osc_ ≈ *f*_inj_ results in the oscillator's phase converging to 0 or 180° (Figure 3e), corresponding to the phenomenon of SHIL, which can also be verified through the quantified fast Fourier transform (FFT) plot in Figure 3f. Notably, during SHIL, the single biristor's waveform appears in two different phases (red and blue) with equal probability (Figure 3d). To further investigate the injection locking behaviors in the InGaAs biristor, output waveforms of the biristor were measured in various *f*_inj_ and voltage amplitude (*V*_inj_) of the injection signal. Given that we set the natural frequency of the InGaAs biristor to ≈250 kHz, both FHIL and SHIL are observed around *f*_osc_ and 2 *f*_osc_, respectively, as shown in Figure 3g. Crucially, the synchronization frequency range exceeds the device‐to‐device (D2D) variability (shown in Figure S5, Supporting Information) of the InGaAs biristors. In prior work on Si biristors with a similar structure to the InGaAs biristor, it has already been demonstrated that even when scaled down to the nanoscale on an 8‐inch wafer, the device‐to‐device variation remains minimal.^[^ ^22^ ^]^ This indicates a capacity for consistent performance and highlights that the InGaAs biristor is an excellent candidate for large‐scale Ising sovlers. Additionally, when the injection voltage is below a certain threshold voltage, the phase of the biristor appears random and unrelated to the injection signal, as shown in Figure 3h. However, once the injection voltage surpasses this threshold, locking occurs at a stable phase. Furthermore, as *V*_inj_ increases, the dispersion of the oscillator phase narrows, indicating much stronger locking. However, strong phase locking might lead the Ising problem, which is intended to be resolved, toward a local minimum, thereby compromising the quality of the solution.^[^ ^23^ ^]^

**Figure 4:**
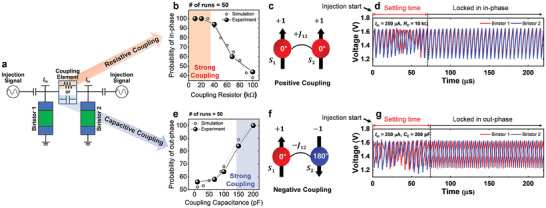
a) The schematic for measuring two coupled InGaAs biristor‐based oscillators utilizing a resistor/capacitor. b) The probability of achieving in‐phase coupling as a function of the coupling resistor (*R*_C_). c) The parallel spin configuration observed during resistive coupling. d) The output waveforms from the two InGaAs biristor‐based oscillators coupled with an *R*_C_ of 10 kΩ, were recorded after the start of injection. e) The probability of achieving out‐of‐phase coupling as influenced by the coupling capacitor (*C*_C_). f) The anti‐parallel spin configuration observed during capacitive coupling. g) The output waveforms from the two InGaAs biristor‐based oscillators coupled with a *C*_C_ of 200 nF, were recorded after the start of injection.

The oscillators must be interconnected through coupling elements to mimic Ising spins. For two coupled Ising spins, there exist two types of coupling: negative (*J*_ij_ = − 1) and positive (*J*_ij_ = + 1) coupling. The schematic for the measurement is presented in **Figure** **4**a, where the coupling trends of phase locking were investigated by adjusting the values of resistors and capacitors. The detailed measurement setup is presented in Figure S6 (Supporting Information). Initially, the coupling trends between two oscillators were examined using resistors. When the value of the resistor was relatively high (>100 kΩ), the phase trends of the two oscillators appeared randomly as in/out. As the resistance decreased, the coupling strengthened, and an in‐phase trend became more pronounced, corresponding to positive *J* where spins are parallel. The waveform measured during the in‐phase trend is shown in Figure 4d, using a resistance of *R*_C_ = 10 kΩ. The results showed that the two phases locked in‐phase configuration with few oscillations after injection started. Conversely, when investigating the coupling trend between two oscillators using capacitors, it was found that at relatively low values (<150 pF), the trend appeared random. However, as the capacitance increased, an out‐of‐phase trend became stronger, corresponding to negative *J* where spins are anti‐parallel. The waveform measured during the out‐of‐phase trend is displayed in Figure 4g, using a capacitance of *C*_C_ = 200 pF. Similar to the resistance measurements, it was observed that the two phases locked out‐of‐phase with few oscillations after injection, just like with the resistance coupling. The trend of coupling behavior based on the values of resistors and capacitors was confirmed to be similar through both measurements and SPICE (Simulation Program with Integrated Circuit Emphasis) simulations. Additionally, the synchronization dynamics of coupled relaxation oscillators using coupling capacitors and resistors are theoretically well described in the work.^[^ ^24^ ^]^

### 2.3 Solving MaxCUT Using InGaAs Biristor‐Based Ising Solver

**Figure 5:**
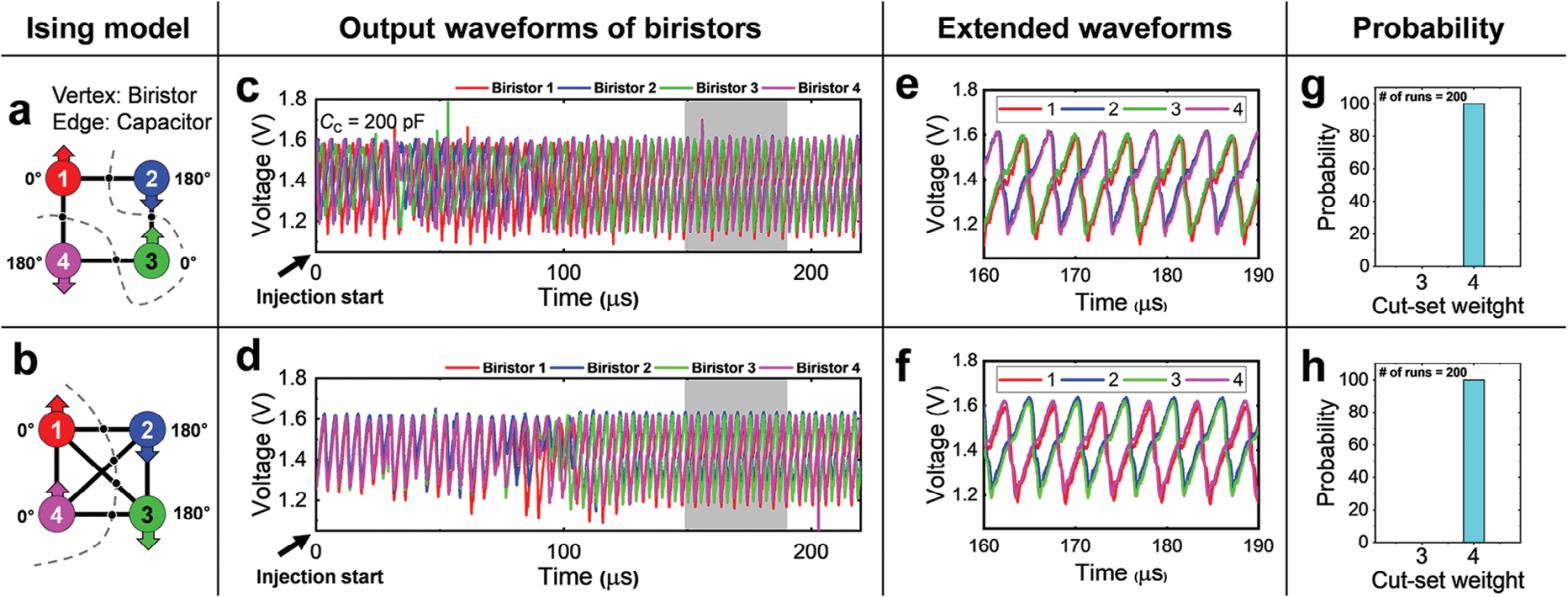
4‐node MaxCUT model with a) 4 edges and b) 6 edges. In these models, each vertex represents an InGaAs biristor, and each edge represents a coupling capacitor. The dotted lines represent the solutions to each MaxCUT problem. c,d) Measured output waveforms of the InGaAs biristor‐based oscillators corresponding to each MaxCUT problem. e,f) An enlarged view of the output waveforms in sections c and d, focusing on the grey areas. g,h) The probability of success after running each MaxCUT problem 200 times.

From the above understandings, we explored the potential of an InGaAs biristor‐based Ising solver for addressing MaxCUT problems. The MaxCUT problem is one of the famous NP‐hard problems that can be directly mapped onto the Ising model.^[^ ^25^ ^]^ The formulation of MaxCUT can be described as follows: given an unweighted graph *G*(*V, E*), where *V* represents the vertices of the graph and *E* denotes the edges between them, the solution to the MaxCUT problem involves dividing *V* into disjoint partitions *V*_1_∪*V*_2_ = *V* in such a manner that the size of the set *E*′ = ((*u*,*v*)∣*u* ∈ *V*_1_, *v* ∈ *V*_2_) is maximized. The value of the cut can be expressed as:

(3) $$C=\sum_{i<j}w_{i,j}\;\frac{\left(1-x_{i}x_{j}\right)}{2}=\frac{1}{2}\sum_{i<j}w_{i,j}\;-\;\frac{1}{2}\sum_{i<j}w_{i,j}x_{i}x_{j}$$

where *w_i,j_* = 1 if (*i,j*) ∈ *E* and 0 otherwise. This value *C* can be redefined in terms of the Ising Hamiltonian *H*(σ) in (1) as:

(4) $$C=\frac{1}{2}\;\sum_{i<j}w_{i,j}\;-\;\frac{1}{2}H\;\left(\sigma \right)$$

with *J*_i,j_ = − *w*_i,j_ = − 1 and zero local magnetic coefficients (*h*_i_). It's important to note that the first term is a constant, and by minimizing the Ising Hamiltonian *H*, the value of cut *C* is maximized. We evaluated the InGaAs biristor‐based Ising solver on a graph *G*(*V*, *E*) with *V* = 4 and *E* = 4, 6 as shown in **Figure** **5**. For the graph with *E* = 4 (Figure 5a), the ground state configuration when the nodes 1 to 4 are arranged in sequence is either (↑↓↑↓) or (↓↑↓↑). The experiments were conducted using an experimental setup, after the injection locking signal was introduced, it was observed that biristors 1 and 3 shared the same phase, while biristors 2 and 4 were out‐of‐phase relative to biristor 1, as shown in Figure 5c,e. Similarly, for *E* = 6 (Figure. 5b), it was confirmed that a solution was reliably reached after a few oscillation cycles (Figure 5d,f), with all 200 runs successfully attaining the ground state (Figure 5g,h). This success can be attributed to the small number of nodes in the MaxCUT problem, which results in fewer local minima, allowing for the achievement of a global solution without the need for a specialized annealing scheme. To analyze a wider range of effects, it will be necessary to connect and study a larger number of oscillators as part of further work.

**Figure 6:**
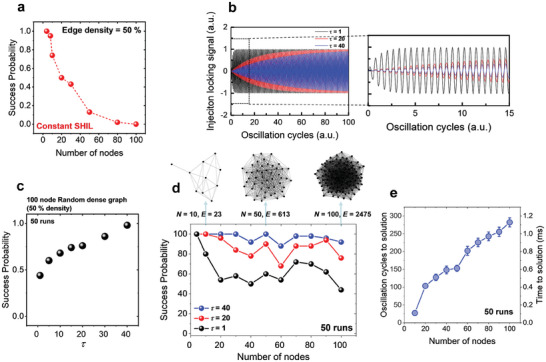
a) The success probability in solving MaxCUT problems with 50% connectivity across varying problem sizes using a constant injection locking scheme. b) The various injection locking schemes corresponding to τ values of 1, 20, and 40. The extended figure is shown on the right. c) Success probability of solving the 100‐node MaxCUT problem with 50% connectivity for different τ values in the annealing scheme. d) The success probability of solving MaxCUT problems with 50% connectivity across various problem sizes for the τ value of 1, 20, and 40. e) The average oscillation cycles to solution (time to solution) according to the number of nodes for different problem sizes. The error bars indicate the standard deviation.

To further assess the feasibility and expected accuracy and performance of the InGaAs biristor‐based Ising solver, we proceeded to model the InGaAs biristor‐based oscillators using a sawtooth‐like function on a basis of phase for the coupling. This involved conducting simulations based on stochastic differential equations (SDEs).^[^ ^10^ ^]^ This step aims to expand upon the feasibility of the InGaAs biristor‐based Ising solver and provides a foundation for evaluating its potential in solving such combinatorial optimization problems. The coupling strength and the SHIL strength were set corresponding to the previously measured values. The success probability is defined as the proportion of trials that successfully identified the true ground‐state energy relative to the total number of trials conducted. Here, the benchmark for the true ground state was established based on solutions obtained from the Biq Mac solver,^[^ ^26^ ^]^ which employs a branch‐and‐bound algorithm, running on a digital CPU (Intel(R) Xeon(R) CPU E5‐2630 v3 @ 2.40 GHz). Additionally, the graphs for testing were generated using the Rudy graph generator, which features a diverse number of nodes and varying edge densities.^[^ ^27^ ^]^ First, we consider the former constant SHIL scheme, keeping the voltage amplitude of SHIL constant throughout the simulation. Initially setting the edge density to 50% for various random dense graphs (with 4‐node graphs having 6 edges, and starting from 8 nodes, maintaining a 50% density), success rates were near 100% up to *V* = 8. However, for *V* = 10 and beyond, success rates significantly dropped and for graphs with more than 100 nodes, the trials failed to reach the best cut solution of the digital CPU's algorithm even once, as shown in **Figure** **6**a. This indicates that in the presence of a large number of nodes, which creates a vast energy landscape, the oscillator network and constant SHIL alone are insufficient for escaping local minima. SHIL primarily serves to force the phases into two distinct states, but it does not inherently guide the system toward lower‐energy solutions. Additionally, providing a constant SHIL signal freezes the network and causes it to become stuck in a local minimum. This suggests the necessity for additional annealing processes, similar to a thermal annealing process where the gradual reduction in temperature correlates with a decrease in stochastic noise within the system, to effectively navigate the solution space.^[^ ^28^ ^]^ In oscillator‐based Ising machines, the annealing process can involve varying the amplitude of the SHIL signal over time or injecting additional white noise to assist in solving combinatorial optimization problems.^[^ ^29^ ^]^ In this work, we aimed to investigate the effectiveness of an annealing scheme that involves changing the amplitude of SHIL over time.

To investigate the effect of SHIL on solution quality over time, particularly within an annealing scheme, we applied the following formula to the injection voltage of the SHIL over time^[^ ^11a^ ^]^:

(5) $$V_{inj}\;\left(t\right)=V_{inj,0}\;\left(1-e^{-\frac{t}{\tau }}\right)$$

where *t* is the oscillation cycle and *V*_inj_ represents the amplitude of the injection locking signal applied to the InGaAs biristors. The scheme of the injection locking signal depending on *τ* is illustrated in Figure 6b. It is observed that for *τ* = 1, the maximum is reached quickly, whereas for *τ* = 40, the maximum is approached nearly at the end of the annealing scheme. Utilizing this annealing scheme for a problem with *V* = 100 and edge density = 50%, experiments were conducted varying *τ*, and the results in Figure 6c showed an increase in success probability as *τ* increased, with *τ* = 40 displaying a success probability close to 100% for 100 nodes. The evolution of phases for 100 oscillators and the cut‐set weight and Ising Hamiltonian for 50 runs are depicted in Figure S7 (Supporting Information). It shows that as the cut‐set reaches the maximum, the energy goes to the minimum, ground state. Moreover, adjusting the annealing scheme for *τ* = 1, 20, 40 and examining the outcomes for various nodes at edge density = 50%, it was found that while the success probability exponentially decreases with an increase in node count for *τ* = 1, the decrease in success probability is significantly less pronounced as *τ* increases, even with more nodes (Figure 6d). This suggests that a slower annealing process, represented by a higher *τ*, can dramatically enhance the solver's ability to find the ground state, thereby maintaining higher success probabilities across larger node counts. Additionally, as the number of nodes increases, leading to a greater number of local minima, it can be observed in Figure 6e that the time required to reach a solution also increases. However, the principle of voltage oscillation in biristors, driven by impact ionization, is inherently random due to stochastic carrier generation. As such, utilizing biristors can produce noise arising from their stochastic characteristics while maintaining low device variability as each component oscillates.^[^ ^22^ ^]^ Therefore, it is anticipated that employing InGaAs biristors as Ising hardware solvers can accelerate problem‐solving, even as the number of nodes grows and local minima become more prevalent, thanks to their noise‐inherent properties.

### 2.4 Performance Benchmark with Other Ising Solvers

**Table** **1** compares various Ising solvers based on key performance metrics for solving 100‐node, random dense MaxCUT problems. This comparison encompasses principal performance indicators such as time to solution and energy to solution. Furthermore, to show the suitability of Ising solvers for large‐scale and high‐density applications, metrics for BEOL‐compatibility and scalability are included. OIMs are noted for their superior performance compared to conventional CPUs and CIMs, owing to their synchronized update scheme. They also demonstrate low power consumption due to utilizing the phase of oscillators as spins. Among OIMs, the InGaAs biristor is distinguished by its unique advantages, including a low process temperature below 100 °C and capability for 3D stacking in any circuit configuration via wafer bonding technology. Utilizing single‐crystalline InGaAs with a narrow bandgap, it offers a solution for the high operational voltages of silicon biristors, ensuring energy efficiency and CMOS compatibility, alongside the advantages of low operating voltages typical of IMT devices. Furthermore, as the frequency of oscillators increases, the solution time of the Ising solver decreases, leading to improved performance. The InGaAs biristor, through mesa size scaling and reduction of parasitic capacitance, can achieve further performance improvements as an Ising solver. This positions the InGaAs biristor‐based approach as an important contribution toward developing the ultimate 3D stackable large‐scale Ising hardware, with significant implications for future technological advancements.

**Table 1:** Benchmark of InGaAs biristor‐based Ising solver with other Ising hardwares for solving MaxCUT on 100‐node 50%‐density unweighted graphs.

	Digital[Simulated Annealing]^[^ ^13^ ^]^	D‐wave 2000Q^[^ ^31^ ^]^	CIM^[^ ^32^ ^]^	Ring oscillator^[^ ^12b^ ^]^	Phase transition device^[^ ^13^ ^]^	LC oscillator^[^ ^11a^ ^]^	This work
Architecture	All to all	Sparse (Chimera)	All to all	King's graph	All to all	All to all	All to all
Ising spin	Digital bits	Qubits	Coherent phase light	Oscillator phases	Oscillator phases	Oscillator phases	Oscillator phases
Implementation	CPU	Compound Josephson‐ junction	OPO (Optical parametric oscillator)	Ring oscillator	VO_2_ device (1T + 1R)	LC oscilaltor	Vertical biristor (1R)
Annealing scheme	Classical	Quantum	Coherent computing	Classical	Classical	Classical	Classical
Update mechanism	Asynchronous	Synchronous	Asynchronous	Synchronous	Synchronous	Synchronous	Synchronous
3D Integration (BEOL compatibility)	× (×)	× (×)	× (×)	× (×)	O (‐)	×	O (< 100 °C)
Scalability (Footprint)	×	×(Cryogenic environment)	×(long fiber ^≈^ km)	O (> 100 F^2^)	O (6 F^2^)	×	O (4 F^2^)
Operating Temp.	Room T.	−273.14 °C	Room T.	Room T.	Room T.	Room T.	Room T.
Time to solution	246 ms	> 10^4^ s (N = 55)	2.3 ms	> 23 µs	30 µs	‐	3 µs^a)^
Energy to solution	14.8 J	> 250 MJ	> 460 mJ	924 nJ	76.8 nJ	‐	120 nJ^a)^

^a)^ Calculated for projected oscillation frequency for size scaling.

## 3 Conclusion

In this work, we have successfully demonstrated an oscillator‐based Ising solver, leveraging the unique properties of single‐crystalline semiconductor devices to introduce a novel artificial spin system. This system not only boasts the smallest footprint among current Ising solvers but also ensures power‐efficient operation, a critical advantage in the realm of computational devices. The InGaAs biristor‐based Ising solver, operating with SHIL, facilitates parallel spin operations through capacitor couplings. This approach achieves unparalleled compactness and scalability in terms of area, further enhanced by BEOL compatibility. This compatibility is largely attributed to the innovative use of low‐temperature fabrication processes and wafer bonding techniques, which are pivotal for integrating this technology into existing semiconductor manufacturing ecosystems. The experimental results, supported by rigorous simulations, highlight the solver's robustness and effectiveness, particularly in addressing the MaxCUT problem—one of the most pertinent NP‐hard problems. The success of this venture opens new avenues for research and development in the field of quantum computing and artificial intelligence, promising a future where complex COPs can be addressed more efficiently and with greater scalability. We anticipate further breakthroughs, including the scaling of coupling elements between oscillators and the development of more efficient architecture arrays, which will enhance the solver's performance and expand its applicability across a broad spectrum of industries and applications.

## 4 Experimental Section

### Epitaxial Growth

To obtain a high‐crystal quality InGaAs active layer, it was we grown on an InP substrate using metalorganic vapor phase epitaxy (MOVPE) while maintaining a negligible lattice mismatch. To achieve a high doping concentration in the collector and emitter of the InGaAs, tellurium (Te)‐doped InGaAs was introduced using a diethyltelluride (DETe) precursor. Furthermore, for the active layer transfer through wafer bonding, a 200 nm InGaAs and a 30 nm InP etch stop was inserted between the active layer and the substrate, The structure of this epitaxy is described in Supplementary Figure S1 (Supporting Information).

### Device Fabrication

After preparing an epitaxially grown InGaAs wafer and a SiO_2_ (1µm)/Si wafer, they were cleaned with acetone, methanol, and deionized water (DI) in a sonication bath. The InGaAs wafer was further cleaned using an NH_4_OH:DI = 1:1 (v/v) solution to eliminate the native oxide. Subsequently, both the InGaAs wafer and the SiO_2_/Si wafer were immediately loaded into an e‐beam evaporator to deposit a contact and bonding metal layer of molybdenum (Mo)/gold (Au) with thicknesses of 20/25 nm, respectively. Following the deposition, the wafers underwent an Ar plasma treatment using a reactive ion etcher (RIE) to prepare them for wafer bonding. The bonding process was then carried out in a 4‐inch wafer bonder at 100 °C for 4 h. After the bonding process, the InP substrate was subjected to wet etching in an HCl solution at room temperature for 50 min. Subsequently, the InGaAs substrate was removed through wet etching with an H_3_PO_4_:H_2_O_2_:DI = 1:1:5 solution for 30 s. To obtain the InGaAs channel, the InP etch stop layer was eliminated using a 45‐s wet etch with an H_3_PO_4_:HCl = 3:1 solution. Next, a positive photoresist (PR) was patterned on the InGaAs layer, and an H_3_PO_4_:H_2_O_2_:DI = 1:1:10 solution was used to etch the InGaAs layer, defining the precise mesa area. The underlying Mo/Au/Mo bonding metal was also defined for device isolation through PR patterning followed by wet etching and RIE dry etching. Subsequently, the native oxide was removed using an NH_4_OH: DI = 1:1 (v/v) solution, followed by sulfur passivation treatment in an (NH_4_)_2_S_x_:DI = 1:10 solution for 10 minutes, which is known to effectively reduce defects in the InGaAs layer.^[^ ^30^ ^]^ SU‐8 was then patterned to passivate the InGaAs, and finally, Mo/Au (20/70 nm) was deposited for the top metal contact using an e‐beam evaporator and lifted off using acetone. The device fabrication schematic is provided in Supplementary Figure S2 (Supporting Information).

### Measurements

The SIMS profile was measured using a Magnetic Sector SIMS IMS 7f (CAMECA). The TEM images were taken using JEM‐ARM200F (JEOL). The DC *I–V* electrical characteristics of the InGaAs biristors were measured using a 4200A‐SCS parameter analyzer (Keithley). Sinusoidal waveforms used for injection locking were generated by AFG 3022B dual channel arbitrary/function generator (Tektronix) and the waveforms were measured using a TBS 2204B oscilloscope (Tektronix).

### Simulation

SPICE simulations were conducted using HSpice (Synopsys) software. To model the InGaAs biristor, a voltage‐controlled switch was connected in parallel with a measured parasitic capacitor of 700 pF. Furthermore, to demonstrate the hysteresis switching characteristics, parameters such as *V*_th_ = 2.0 V, *V*_h_ = 1.0 V, *R*_on_ = 150 Ω, and *R*_off_ = 10^6^ Ω were utilized. To address the MaxCUT problems through multiple simulation runs, a stochastic differential equation solver was employed within a MATLAB simulation framework to closely replicate the dynamics of the InGaAs biristor‐based oscillator system under injection locking. The fitting of both the coupling strength and the injection locking strength was aligned with the experimental observations in Figures 4 and 5. The magnitude of noise in the simulation model was based on the jitter noise of the InGaAs biristor‐based oscillator.

## Conflict of Interest

The authors declare no conflict of interest.

## Author Contributions

J.P.K conducted the device fabrications, characterizations, and analysis; H.W.K conducted the circuit‐based simulation; J.J,J.P, S.K,J.K, and J.W contributed in the experiment and characterization methodology; S.H.K supervised the project; J.P.K wrote the manuscript.

## Supporting information



Supporting Information

## Data Availability

The data that support the findings of this study are available from the corresponding author upon reasonable request.
