# Supplementary material for: BEOL‐Compatible 4F2 Oscillator Using Vertical InGaAs Biristor for Highly Scalable Monolithic 3D Ising Solver
Source: Small. 2024 Oct 21;20(52):2406822. doi: 10.1002/smll.202406822 (PMC11673444; doi:10.1002/smll.202406822)
Supplement: Supplementary file 1 — Supporting Information [file SMLL-20-2406822-s001.docx]

Copyright WILEY-VCH Verlag GmbH & Co. KGaA, 69469 Weinheim, Germany, 2016.

Supplementary Information

**BEOL-compatible 4F^2^ Oscillator using Vertical InGaAs Biristor for Highly Scalable Monolithic 3D Ising Solver**

*Joon Pyo Kim, Hyun Wook Kim, Jaeyong Jeong, Juhyuk Park, Song-Hyeon Kuk, Jongmin Kim, Jiyong Woo, and Sanghyeon Kim**

**Figure S1.** Schematic structure of the epitaxy used for BEOL compatible InGaAs biristor-based oscillator.

**Figure S2.** Device fabrication process of the InGaAs biristor-based oscillator.

**Figure S3.** Oscillation frequency according to the injection current.

Figure S3 shows that as the input current applied to the collector of the InGaAs biristor increases, the voltage oscillation frequency accelerates.

**Figure S4. Oscillation frequency according to the parasitic capacitor.**

Figure S4 illustrates the measured oscillation frequency of the InGaAs biristor concerning the parasitic capacitance (*C*_Par_) of 1, 10, and 100 nF, indicated by closed circles, and without any parasitic capacitance, indicated by an open circle. Despite not connecting any additional *C*_Par_ for the measurements, the fact that the oscillation frequency does not significantly increase suggests the presence of inherent parasitic capacitance within the measurement setup. The trend line indicates that this parasitic capacitance is approximately 700 pF. In addition, by scaling down the *C*_Par_, the frequency of the biristor can be increased. Therefore, if we can scale down the size of the biristor and its parasitic capacitance, we expect that the frequency of the biristor will be comparable to that of a moderate CMOS ring oscillator.

**Figure S5. Distribution of the oscillation frequency of the fifty InGaAs biristors during the injection current of 250 μA**.
The measured oscillation frequency of the fifty InGaAs biristors indicates a device variability of approximately 2.3%. Given that the injection locking window, as illustrated in **Figure 3g**, is broader than the device variability, this suggests that the InGaAs biristor is a suitable candidate for a large-scale Ising solver capable of stable operation, thanks to its minimal device variability.

**Figure S6. The experimental measurement setup for the InGaAs biristor-based Ising solver.** (a) The setup includes a function generator, oscilloscope, Keithley 4200 semiconductor parameter analyzer, and a custom printed circuit board (PCB) equipped with the fabricated InGaAs biristor array. (b) The custom PCB features SMA connectors for external equipment connections and sockets for inserting injection and coupling capacitors. The InGaAs biristor array is mounted at the center of the PCB, with the metal pads of the biristors wirebonded and connected to the PCB pads. (c) An optical microscope (OM) image showcases the fabricated InGaAs biristors, providing a close-up view of their structure and layout.

**Figure S7. The numerical simulation result of 50 trials of the InGaAs-biristor-based Ising solver for the 100-node MaxCUT problem with 50 % connectivity.** (a) The evolution of the phases of the 100 oscillators in a single run as the solver attempts to address the problem, offers a visual representation of how the system dynamically seeks the optimal solution over time. (b) The normalized cut-set weight and (c) the Ising Hamiltonian across the 50 trials are depicted, with a grey line representing all trials and a black line highlighting the simulation result for the trial with the best-cut outcome. The results indicate the efficiency and effectiveness of the solver in partitioning the graph to maximize the cut-set weight. In addition, the graph provides a statistical overview of the solver's ability to minimize the Ising Hamiltonian, which directly correlates with maximizing the value of the cut in the MaxCUT problem.

**Supplementary Note 1**

In the study of Ising machines based on oscillators, it's essential to understand the behavior of oscillator networks when subjected to external disturbances, especially through the phenomenon of injection locking. Adler's equation plays an important role in understanding how oscillators react to external sinusoidal injection locking signals, showing the evolution of the phase difference between an oscillator and the external signal. When expanding this framework to include non-linear oscillators affected by periodic injection locking signals, the generalized version of Adler's equation (referred to as Gen-Adler) is relevant^[1]^. This equation allows for the prediction of multiple stable phase-locked states within the oscillator network.

For an oscillator with a natural frequency *f*_osc_ disturbed by a periodic external frequency *f*_inj_, similar to what is known as first-harmonic injection locking (FHIL), the Gen-Adler equation provides a means to predict its phase response:

| $\frac{d\Delta\theta(t)}{dt}=-\left( f_{inj}-f_{osc} \right)+f_{osc}g(\Delta\theta\left( t \right))$ | (1) |
| --- | --- |

Here, Δ$\theta(t)$ = $\theta_{osc}\left( t \right)-\theta_{in}(t)$ represents the phase difference between the oscillator and the injection signal, with *g*(.) being a periodic function termed the perturbation projection vector (PPV), which is influenced by both the oscillator’s characteristics and the injection signal. When the oscillator is locked by an injection signal, Δ$\theta(t)$ becomes a constant, leading to $\frac{d\Delta\theta(t)}{dt}=0$, simplifying the (1) to:

| $\frac{f_{inj}-f_{osc}}{f_{osc}}=g(\Delta\theta\left( t \right))$ | (2) |
| --- | --- |

This simplified equation enables the prediction of injection locking's extent and the phase difference between the oscillator and the external signal, as illustrated in **Figure S8a.**

Furthermore, when the oscillator faces an external injection signal approximately double its fundamental frequency, known as sub-harmonic injection locking (SHIL), the Gen-Adler equation modifies to:

| $\frac{d\Delta\theta(t)}{dt}=-\left( {\frac{1}{2}f}_{inj}-f_{osc} \right)+f_{osc}g(\Delta\theta\left( t \right))$ | (3) |
| --- | --- |

When locked by the injection signal, $\frac{d\Delta\theta(t)}{dt}=0$, it follows that:

| $\frac{{\frac{1}{2}f}_{inj}-f_{osc}}{f_{osc}}=g(\Delta\theta\left( t \right))$ | (4) |
| --- | --- |

According to this equation, it becomes apparent that stable phases lock at two of the four intersections, indicative of stable phase-locked states, as depicted in **Figure S8b**. These states are separated by a phase difference of 180°. The Gen-Adler framework is a potent tool for forecasting and decoding the dynamics of injection locking within oscillators.

**Figure S8. Illustration of the Generalized Adler’s equation under injection locking.** (a) first-harmonic injection locking (b) sub-harmonic injection locking.

**References**

[1] P. Bhansali, J. Roychowdhury, in *2009 Asia and South Pacific Design Automation Conference* IEEE, **2009**, 522-527.
